# Coagulation Factors and White Matter Hyperintensities in Middle‐Aged Women With and Without Migraine and Ischemic Stroke

**DOI:** 10.1111/ene.70063

**Published:** 2025-02-28

**Authors:** Mariam Ali, Nelleke van der Weerd, Hendrikus J. A. van Os, Annelise E. Wilms, Ghislaine Holswilder, Katie M. Linstra, Thijs W. van Harten, Suzanne C. Cannegieter, Bob Siegerink, Bart J. M. van Vlijmen, L. Renee Ruhaak, Nyika D. Kruyt, Mark C. Kruit, Antoinette Maassen Van Den Brink, Gisela M. Terwindt, Marieke J. H. Wermer, The CREW Consortium

**Affiliations:** ^1^ Neurology, Leiden University Medical Centre (LUMC) Leiden the Netherlands; ^2^ Human Genetics, LUMC Leiden the Netherlands; ^3^ Public Health & Primary Care, LUMC Leiden the Netherlands; ^4^ Radiology, LUMC Leiden the Netherlands; ^5^ Neurology, Massachusetts General Hospital Harvard Medical School Boston USA; ^6^ Clinical Epidemiology, LUMC Leiden the Netherlands; ^7^ Internal Medicine, Thrombosis & Hemostasis, LUMC Leiden the Netherlands; ^8^ Clinical Chemistry & Laboratory Medicine, LUMC Leiden the Netherlands; ^9^ Internal Medicine Rotterdam the Netherlands; ^10^ Neurology, University Medical Centre Groningen Groningen the Netherlands

**Keywords:** coagulation factors, ischemic stroke, migraine, white matter hyperintensities, women

## Abstract

**Background:**

Migraine increases the risk of ischemic stroke and white matter hyperintensities (WMH), especially in women. Underlying shared mechanisms may include endothelial activation and hypercoagulability. We assessed these markers in middle‐aged women with and without migraine and ischemic stroke to explore their role in WMH development.

**Methods:**

We cross‐sectionally measured fibrinogen and von Willebrand factor antigen (VWF:Ag) levels as markers of endothelial activation, and factor (F)IX and FXI activity (FXI:C) as markers of hypercoagulability in four groups of women aged 40–60 years with (1)ischemic stroke, (2)migraine, (3)both ischemic stroke and migraine, and (4)no stroke or migraine. Multivariable linear regression estimated mean differences in coagulation factor levels between groups 1 and 3, with group 4 as the reference.

**Results:**

Among 166 women (mean age:51 years), (1) 45 had ischemic stroke, (2) 38 had migraine, (3) 48 had both, and (4) 35 had no stroke or migraine. Mean fibrinogen, FIX, and FXI:C levels were similar in all groups compared with group 4. VWF:Ag levels were higher in the ischemic stroke with migraine group (170 [57]%; adjusted *β* = 31%; 95% CI = 6%–56%) compared with group 4, but not in the other groups. There was no association between coagulation factors and WMH volume in any group.

**Conclusions:**

In women with both stroke and migraine, VWF:Ag levels were increased, while fibrinogen, FIX, and FXI:C were not. This suggests that endothelial activation might be more relevant than a procoagulant state alone in the pathophysiology of ischemic stroke in women with migraine. Coagulation factors did not seem to be related to WMH volume, suggesting other mechanisms may be involved in their development.

## 1 Introduction

Women with migraine, especially with aura, have an increased risk of ischemic stroke [1]. White matter hyperintensities (WMH) are also more common in women with migraine with aura, suggesting small‐vessel involvement in the pathophysiology linking migraine and ischemic stroke [2, 3]. Understanding the pathophysiology of WMH in women with migraine is important for clarifying how migraine contributes to microvascular damage and stroke risk.

The risk of ischemic stroke is particularly elevated in women under 45 years with migraine, likely due to nonatherosclerotic mechanisms that differ from those in men [1]. This risk is not explained by conventional cardiovascular risk factors [4]. Additionally, frequent migraine attacks increase the risk of WMH [3]. Several prothrombotic genetic polymorphisms have been linked to migraine, ischemic stroke, and WMH, suggesting hypercoagulability plays a role in the pathophysiology of these conditions [5, 6, 7, 8, 9].

The main hypotheses linking migraine with stroke and WMH are endothelial activation and hypercoagulability, which in part overlap [10, 11]. Endothelial activation involves the stimulation of inflammatory and procoagulant pathways, resulting in a prothrombotic and inflammatory state [11]. Transient endothelial activation during migraine attacks is thought to play a key role in the migraine‐ischemic stroke relationship [12, 13, 14, 15]. This process can trigger the coagulation pathway, leading to clot formation through the release of proteins such as von Willebrand factor (VWF) and fibrinogen [13]. This activation contributes to ischemic stroke and WMH by causing inflammation, thrombosis, impaired cerebral blood flow regulation, increased blood–brain barrier permeability, and activation of the intrinsic coagulation pathway [16, 17]. Hypercoagulability, on the other hand, refers to an increased tendency for clot formation due to elevated coagulation factor levels. While women with migraine often show higher platelet activity and elevated fibrinogen and VWF levels compared with those without migraine, this association is inconsistent [9, 10, 18, 19, 20].

In this study, we first assessed whether coagulation factors, including those related to endothelial activation, are increased in middle‐aged women with both ischemic stroke and migraine compared with those without stroke or migraine, to examine the role of coagulation and endothelial involvement in migraine‐ischemic stroke pathophysiology. We also included women with ischemic stroke alone and migraine alone to explore a potential supra‐additive effect. Second, we investigated whether these coagulation factors are associated with WMH volume.

## 2 Methods

### 2.1 Study Design and Participants

We included women aged 40–60 from two cross‐sectional studies conducted at the Leiden University Medical Center (LUMC) between October 2016 and December 2021, following identical protocols: the Cardiovascular RiskprofilE in Women—MIcrovascular STatus (CREW‐MIST) and WHIte matter lesions in young to middle‐aged women with Stroke, PreEclampsia and migRaine (WHISPER). This age range was selected to ensure a relatively homogeneous population at risk for stroke, while minimizing the confounding effects of age‐related cardiovascular risk factors and atherosclerosis. From these studies, we constructed four groups: (1) women with a history of ischemic stroke, (2) women with migraine with aura, (3) women with a history of both ischemic stroke and migraine, and (4) women with no history of ischemic stroke or migraine.

Groups 1 and 3 were derived from CREW‐MIST, which recruited women from in‐ and outpatient neurology clinics of the LUMC and University Medical Center Utrecht, as part of the CREW Consortium. All eligible patients were randomly invited to participate. Additional recruitment was conducted through nationwide advertising by the Dutch Heart Foundation, and all participants were referred to the LUMC for data collection and MRI scans. Ischemic stroke was defined as an acute neurological deficit lasting more than 24 h with corresponding lesions on CT or MRI, based on medical records, discharge letters, and neuroimaging reports, occurring at least 3 months before inclusion. Stroke subtypes were classified according to the TOAST criteria (Trial of ORG 10172 in Acute Stroke Treatment) [21]. Women in group 3 had a history of migraine (with or without aura), diagnosed according to the International Classification of Headache Disorders‐3 (ICHD‐3) criteria [22]. Information on migraine characteristics and medication use was collected through questionnaires.

Groups 2 and 4 were derived from the WHISPER study. Group 2 included women with migraine with aura (no participants with migraine without aura were included) from the Leiden University Migraine Neuro‐Analysis (LUMINA) database of the LUMC. This group was restricted to those experiencing ≥ 1 attack per month, as monitored using electronic headache diaries [23]. The LUMINA database comprises adults aged 18–80 years diagnosed with migraine according to the ICHD‐3 criteria and assessed using the validated LUMINA questionnaire [23]. All eligible patients in this database were randomly invited to participate. Group 4 was recruited through nationwide advertising by the Dutch Heart Foundation. Both CREW‐MIST and WHISPER excluded women with other neurological disorders (e.g., reversible cerebral vasoconstriction syndrome), systemic diseases involving demyelinating or inflammatory white matter brain lesions, retinal, spinal, or cerebral venous infarctions, current pregnancy, illnesses compromising study participation, contraindications for MRI, or insufficient proficiency in Dutch or English. Patients on anticoagulants at the time of blood withdrawal were excluded from analyses.

During a 1‐day visit, participants completed questionnaires on demographics and medical history, underwent blood withdrawal, and had a 3 T brain MRI. For participants with a history of migraine, the visit was postponed if a migraine attack occurred on that day.

### 2.2 Coagulation Factors

We selected four coagulation factors for analysis: fibrinogen and VWF antigen (VWF:Ag) as markers of endothelial activation, and factor IX (F) IX and FXI activity (FXI:C) as markers of hypercoagulability, based on their established relevance to thrombotic risk [24]. Plasma levels of FXI:C and VWF:Ag were measured in duplicate, whereas fibrinogen and FIX were measured in single. Blood samples were collected in vacuum citrate tubes, centrifuged at 2350 RCF for 10 min at 20°C, aliquoted, and stored at −80°C.

FXI:C and VWF:Ag levels were measured using the ACL‐Top 550 Analyzer. FXI:C levels were determined with specific activated partial thromboplastin time assays. Automated latex‐enhanced immunoassays were used to measure VWF with VWF:Ag reagent. Fibrinogen and FIX levels were assessed with targeted protein mass spectrometry (Supporting Information).

### 2.3 WMH Volume Assessment on MRI

Participants were scanned using a 3T MRI scanner (Philips, Best, the Netherlands). WMH volume was initially identified using a semiautomatic method, after which true WMH was confirmed by a human rater. Two independent raters (AEW and NvdW) scored volumes supervised by a senior neuroradiologist (MCK, 20 + years of experience in the field). Further details on MRI acquisition and WMH volume analysis are in the Supplemental Methods.

### 2.4 Statistical Analysis

We performed descriptive statistics for baseline characteristics and visually inspected the distributions of coagulation factors and total WMH volume for normality. First, we conducted linear regression analyses adjusting for age, BMI, smoking (ever), menopause, and oral contraceptive use. Regression coefficients (*β*) with corresponding 95% confidence intervals (CIs) were calculated to estimate mean differences in coagulation factor levels between groups 1 and 3, using group 4 as the reference. Second, we analyzed the association between coagulation factor levels and total WMH volume within each group, both visually (with evaluation on a logarithmic scale to ease interpretation) and using linear regression models with the same covariables. For ischemic stroke patients, the model was further adjusted for TOAST classification.

### 2.5 Ethics Statement

The Medical Ethics Committee Leiden—Den Haag—Delft approved the study protocols for CREW‐MIST (P15.384) and WHISPER (P18.130). In accordance with the Declaration of Helsinki, all patients provided written informed consent.

### 2.6 Reporting Standards

This manuscript follows the STROBE reporting guideline.

## 3 Results

A total of 166 participants were included: 45 (27%) had a history of ischemic stroke (group 1, mean age 51 years), 38 (23%) had migraine with aura (group 2, mean age 51 years), 48 (29%) had both ischemic stroke and migraine (group 3, mean age 51 years, with 26 [54%] with aura), and 35 (21%) had neither ischemic stroke nor migraine (group 4, mean age 52 years) (Table 1).

**TABLE 1:** Baseline characteristics of participants.

	1. Ischemic stroke only (+/−) (*n* = 45)	2. Migraine with aura only (−/+) (*n* = 38)	3. Ischemic stroke and migraine (+/+) (*n* = 48)	4. No ischemic stroke or migraine (−/−) (*n* = 35)
Age at visit, years (mean (SD))	50.6 (5.0)	51.3 (4.9)	51.3 (5.3)	52.2 (5.0)
Age at stroke, years (mean (SD))	46.6 (6.2)	—	45.2 (6.6)	—
Time between stroke and research visit, years (mean (SD))	3.9 (3.2)	—	5.7 (5.9)	—
White ethnicity	40/42 (95.2)	27/29 (93.1)	38/41 (92.7)	30/32 (93.8)
Body mass index, kg/m^2^ (mean (SD))^a^	26.9 (4.4)	25.3 (4.2)	27.1 (4.6)	25.1 (4.1)
History of intracerebral hemorrhage or TIA	4 (8.9)	—	5 (10.4)	—
Cardiovascular risk factors				
Diabetes mellitus	0/45 (0)	1/35 (2.9)	2/48 (4.2)	0/35 (0)
Hyperlipidemia	36/45 (80.0)	7/38 (18.4)	38/48 (79.2)	2/35 (5.7)
Hypertension	27/45 (60.0)	12/38 (31.6)	30/48 (62.5)	7/35 (20.0)
Smoking	5/43 (11.6)	4/29 (13.8)	3/41 (7.3)	2/32 (6.3)
Alcohol use	28/43 (65.1)	19/29 (65.5)	27/41 (65.9)	26/32 (81.3)
Antithrombotic medication				
Antiplatelets	40/43 (93.0)	4/38 (10.5)	47/48 (97.9)	0/35 (0)
Vitamin K antagonist	2/43 (4.7)	1/38 (2.6)	1/48 (2.1)	0/35 (0)
Prophylactic LMWH	6/43 (14.0)	4/38 (10.5)	3/48 (6.3)	0/35 (0)
Gynecological parameters				
Postmenopausal	19/45 (42.2)	19/38 (50.0)	20/48 (41.7)	16/33 (48.5)
Current use of combined or progestin‐only oral contraceptives	1/45 (2.2)	5/38 (13.2)	1/48 (2.1)	2/35 (5.7)
Migraine characteristics				
Migraine with aura	—	38 (100)	26 (54.2)	—
Migraine without aura	—	0 (0)	22 (45.8)	—
Average lifetime migraine attack frequency p/year				
1–12 (i.e., an average of 1 attack p/month)	—	9 (23.7)	29 (60.4)	—
13–54 (i.e., an average of 1–4 attacks p/month)	—	29 (76.3)	15 (31.3)	—
> 54 (i.e., several attacks p/week)	—	0 (0)	4 (8.3)	—
Stroke cause according to the TOAST criteria				
Large artery atherosclerosis	12/45 (26.7)	—	9/46 (19.6)	—
Cardioembolic	2/45 (4.4)	—	4/46 (8.7)	—
Small vessel occlusion	10/45 (22.2)	—	9/46 (19.6)	—
Other cause	8/45 (17.8)	—	2/46 (4.3)	—
Undetermined cause	13/45 (28.9)	—	22/46 (47.8)	—

*Note:* Data are *n*/*N* (%), unless otherwise specified.

Abbreviations: LMWH, low molecular weight heparin; SD, standard deviation; TOAST, trial of ORG 10172 in acute stroke treatment.

^a^ Missing values, *n* (%): 7 (14.6%) vs. 3 (6.7%) vs. 9 (23.7%) vs. 3 (8.6%).

No women with migraine‐induced stroke were included in the study. Baseline characteristics and traditional cardiovascular risk factors were similar between ischemic stroke groups but differed substantially from those in group 2 (migraine with aura) and group 4 (no history of ischemic stroke or migraine). Stroke etiologies also differed substantially between groups 1 and 3 (Table 1).

### 3.1 Coagulation Factors

In women of group 4 (no history of stroke or migraine), the mean (SD) levels of fibrinogen, VWF:Ag, FIX, and FXI:C were 3.7 (0.7) g/L, 140 (32)%, 1.2 (0.3) IU, and 1.1 (0.2) IU/mL, respectively. There were no statistically significant mean differences in fibrinogen, FIX, or FXI:C levels between groups 1 (ischemic stroke), 2 (migraine with aura), and 3 (ischemic stroke and migraine) compared with the reference group (group 4: no stroke, no migraine) (Table 2). The mean VWF:Ag level in group 3 (ischemic stroke and migraine) was 170 (57)%, with an adjusted mean difference of 31% (95% CI, 6%–56%) compared with the reference group. VWF:Ag levels were also slightly elevated in both groups 1 and 2, but these differences were much smaller than in group 3.

**TABLE 2:** Crude and adjusted mean differences in coagulation factor levels (95% CIs) using women with no history of ischemic stroke or migraine (group 4) as the reference.

	Mean (SD) levels	Unadjusted *β* (mean difference with 95% CI)	Adjusted *β* (mean difference with 95% CI)^a^
**Fibrinogen (g/L)**			
1. Ischemic stroke only (+/−) (*n* = 45)	3.38 (0.59)	−0.28 (−0.57 to 0.01)	−0.21 (−0.52 to 0.10)
2. Migraine with aura only (−/+) (*n* = 38)	3.33 (0.75)	−0.34 (−0.68 to 0.01)	−0.35 (−0.70 to 0.01)
3. Ischemic stroke and migraine (+/+) (*n* = 48)	3.30 (0.51)	−0.36 (−0.63 to −0.09)	1.21 (−1.34 to 3.76)
4. No ischemic stroke or migraine (−/−) (*n* = 35)	3.66 (0.72)	Reference	Reference
**FIX (IU)**			
1. Ischemic stroke only (+/−) (*n* = 45)	1.12 (0.23)	−0.05 (−0.18 to 0.07)	−0.01 (−0.15 to 0.12)
2. Migraine with aura only (−/+) (*n* = 38)	1.11 (0.24)	−0.06 (−0.19 to 0.07)	−0.04 (−0.19 to 0.11)
3. Ischemic stroke and migraine (+/+) (*n* = 48)	1.10 (0.21)	−0.07 (−0.19 to 0.05)	−0.05 (−0.18 to 0.07)
4. No ischemic stroke or migraine (−/−) (*n* = 35)	1.17 (0.32)	Reference	Reference
**FXI activity (IU/mL)**			
1. Ischemic stroke only (+/−) (*n* = 45)	1.20 (0.18)	0.05 (−0.03 to 0.14)	0.08 (−0.02 to 0.18)
2. Migraine with aura only (−/+) (*n* = 38)	1.26 (0.20)	0.11 (0.02 to 0.21)	0.12 (−0.02 to 0.23)
3. Ischemic stroke with migraine (+/+) (*n* = 48)	1.19 (0.19)	0.04 (−0.05 to 0.13)	0.07 (−0.03 to 0.15)
4. No ischemic stroke or migraine (−/−) (*n* = 35)	1.14 (0.20)	Reference	Reference
**VWF antigen (%)**			
1. Ischemic stroke only (+/−) (*n* = 45)	151.47 (60.09)	11.92 (−10.32 to 34.15)	15.03 (7.33 to 38.39)
2. Migraine with aura only (−/+) (*n* = 38)	150.60 (53.2)	11.04 (−9.63 to 31.71)	10.30 (2.60 to 33.15)
3. Ischemic stroke and migraine (+/+) (*n* = 48)	170.21 (56.63)	30.65 (9.33 to 51.97)	31.36 (6.22 to 55.70)
4. No ischemic stroke or migraine (−/−) (*n* = 35)	139.56 (32.07)	Reference	Reference

Abbreviations: CI, confidence interval; SD, standard deviation; VWF, von Willebrand factor.

^a^ *β* coefficients were calculated using linear regression models adjusted for age, BMI, smoking (ever), menopause, and use of oral contraceptives.

### 3.2 Coagulation Factors and Total WMH Volume

**FIGURE 1:**
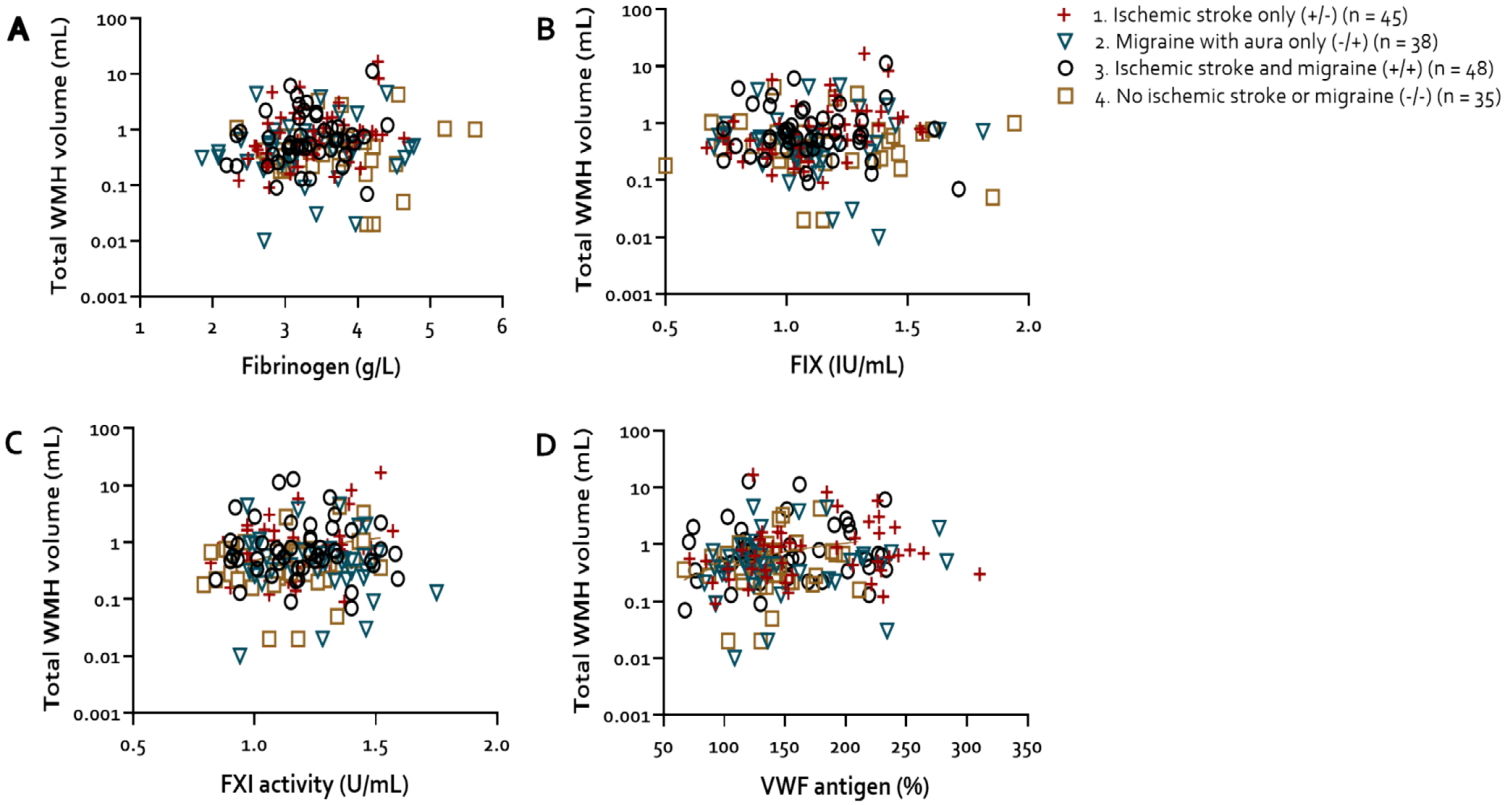
Assocation between coagulation factor levels and total WMH volume. VWF, von Willebrand factor; WMH, white matter hyperintensities.

Total mean (SD) WMH volume was (1) 1.51 (2.80) mL in the ischemic stroke group, (2) 0.81 (1.11) mL in the migraine with aura group, (3) 1.46 (2.52) mL in the ischemic stroke and migraine group, and (4) 0.68 (0.92) mL in the group without stroke or migraine. We found no relation between fibrinogen, VWF:Ag, FIX, and FXI:C levels and total WMH volume in any of the four groups, both visually and with linear regression models (Table 3, Figure 1). The regression models for ischemic stroke patients did not change when additionally adjusted for TOAST classification (data not shown).

**TABLE 3:** Crude and adjusted linear regression coefficients (95% CIs) for the association between coagulation factor levels and total WMH volume within each group.

	Unadjusted *β* (95% CI)	Adjusted *β* (95% CI)
**Fibrinogen (g/L)**		
1. Ischemic stroke only (+/−) (*n* = 45)	−1.36 (−19.29 to 16.57)	4.81 (−7.75 to 5.58)
2. Migraine with aura only (−/+) (*n* = 38)	0.36 (−0.78 to 1.49)	2.64 (−0.72 to 4.00)
3. Ischemic stroke and migraine (+/+) (*n* = 48)	−0.56 (−12.73 to 11.61)	2.44 (−0.15 to 4.99)
4. No ischemic stroke or migraine (−/−) (*n* = 35)	0.29 (−0.18 to 0.69)	0.69 (−0.89 to 2.39)
**FIX (IU)**		
1. Ischemic stroke only (+/−) (*n* = 45)	−5.21 (−9.69 to 6.28)	2.83 (−1.73 to 5.80)
2. Migraine with aura only (−/+) (*n* = 38)	4.53 (2.13 to 6.92)	2.67 (−0.92 to 1.63)
3. Ischemic stroke and migraine (+/+) (*n* = 48)	−10.69 (−24.08 to 12.71)	−1.89 (−0.57 to 4.99)
4. No ischemic stroke or migraine (−/−) (*n* = 35)	0.63 (−1.22 to 2.47)	1.16 (−1.42 to 4.81)
**FXI activity (IU/mL)**		
1. Ischemic stroke only (+/−) (*n* = 45)	4.82 (0.39 to 9.25)	11.16 (−15.23 to 20.56)
2. Migraine with aura only (−/+) (*n* = 38)	−0.65 (−2.47 to 1.17)	−1.72 (−16.29 to 12.84)
3. Ischemic stroke and migraine (+/+) (*n* = 48)	−0.58 (−4.42 to 3.25)	−3.21 (−7.11 to 5.31)
4. No ischemic stroke or migraine (−/−) (*n* = 35)	1.41 (−0.13 to 2.96)	3.03 (−3.69 to 6.12)
**VWF antigen (%)**		
1. Ischemic stroke only (+/−) (*n* = 45)	0.001 (−0.01 to 0.02)	−0.001 (−0.02 to 0.02)
2. Migraine with aura only (−/+) (*n* = 38)	0.002 (−0.01 to 0.01)	0.002 (−0.008 to 0.01)
3. Ischemic stroke and migraine (+/+) (*n* = 48)	−0.002 (−0.02 to 0.01)	−0.003 (−0.02 to 0.01)
4. No ischemic stroke or migraine (−/−) (*n* = 35)	0.006 (−0.004 to 0.02)	0.01 (−0.004 to 0.03)

*Note:* *β* coefficients were calculated using linear regression models adjusted for age, BMI, smoking (ever), menopause, and use of oral contraceptives. The model for ischemic stroke patients was further adjusted for TOAST classification.

Abbreviations: CI, confidence interval; VWF, von Willebrand factor; WMH, white matter hyperintensities.

## 4 Discussion

In this cross‐sectional study, we found higher VWF:Ag levels in all groups—ischemic stroke (group 1), migraine with aura (group 2), and ischemic stroke and migraine (group 3)—compared with the reference group (group 4), with 31% higher levels in the ischemic stroke and migraine group (group 3), statistically significantly higher than in the groups with only stroke (group 1) or only migraine with aura (group 2). No such differences were found for fibrinogen, FIX, or FXI:C. We found no relationship between coagulation factor levels and total WMH volume.

Previous studies on hypercoagulability in ischemic stroke patients with migraine have typically examined individual coagulation factors in small samples (*N* < 200 participants) [10, 20, 25, 26]. Our study, while also constrained by a relatively small sample size, offers a more comprehensive analysis by assessing multiple coagulation factors linked to endothelial activation and hypercoagulability. We found elevated VWF:Ag levels in the ischemic stroke with migraine group, suggesting a possible supra‐additive effect. This combination may result in greater endothelial activation than either condition alone. These findings align with growing evidence of increased endothelial activation in ischemic stroke associated with migraine, particularly in women [11].

Migraine, particularly with aura, is associated with endothelial activation, proinflammatory pathways, and an increased risk of ischemic stroke [11]. Although cortical spreading depolarization, the physiological basis of aura, has been hypothesized to activate the trigeminovascular system and contribute to a procoagulant and inflammatory milieu, direct evidence connecting CSD to endothelial dysfunction remains absent [27]. Instead, endothelial dysfunction may broadly create a procoagulant and inflammatory environment, promoting oxidative stress, impairing endothelial repair, and increasing vascular damage [13]. Our findings support these broader mechanisms, suggesting that migraine may play a potential role in stroke pathophysiology through these pathways.

In addition, neuroinflammatory processes involving cytokines like interleukin‐6 and markers such as C‐reactive protein are common in both migraine and stroke [13]. However, we found no association between VWF:Ag levels and total WMH volume in the ischemic stroke with migraine group [27]. This may reflect the etiology of strokes in these patients, which were mainly due to undetermined causes, including a few cases of arterial dissection and patent foramen ovale. Such mechanisms are less likely to involve hypercoagulability, potentially explaining the observed lack of association with WMH volume [11].

Our study has several limitations. First, the cross‐sectional design limits our ability to establish causal relationships between coagulation factors and WMH development. The lack of longitudinal data also prevents us from assessing how coagulation factor levels and WMH progress over time within the groups. Since migraine onset is typically early in life while ischemic stroke tends to develop later, migraine almost always precedes stroke, making the temporal relationship clear for most individuals with both conditions. However, it remains unclear whether women with migraine who develop WMH early in life are at higher risk of subsequent ischemic stroke compared with those who do not.

Longitudinal studies are necessary to clarify temporal relationships. Second, we focused solely on women due to the much higher prevalence of migraine in this population, which limits the generalizability of our results. This focus is clinically more relevant, as the associations between migraine, ischemic stroke, and WMH are stronger in women, particularly in those with migraine with aura, whereas these associations are more uncertain in men [28]. Third, the small sample size may have reduced our power to detect associations between groups and coagulation factors, as well as between coagulation factors and total WMH volume. Given the exploratory nature of the study and the small sample size, we performed a complete case analysis without adjustments for multiple testing or additional covariables [29]. The small sizes of the migraine with aura group and the group without stroke or migraine further restricted subgroup analyses, such as comparisons between pre‐ and postmenopausal women or migraine subtypes. In the ischemic stroke group, both migraine with and without aura were included due to the limited number of available patients with both conditions. This inclusion introduced heterogeneity when comparing this group with the migraine with aura‐only group. Fourth, while we selected coagulation factors associated with a high risk of thrombosis, we could not measure FVIII, a procoagulant factor strongly associated with venous thrombosis risk due to its central role in hemostasis [24]. However, as FVIII is strongly correlated with VWF, similar results would likely have been found *for FVIII and VWF analyses* [24]. Additionally, we evaluated only VWF:Ag levels without assessing VWF activity or ADAMTS13, which may provide further insights into the role of VWF in thrombosis. Fifth, selection bias may have been introduced as this is a clinic‐based rather than a population‐based study.

Strengths of our study include the use of a cohort with a standardized diagnosis of migraine following ICHD‐3 criteria, along with standardized measurements of WMH volume and plasma samples from two cross‐sectional studies. Additionally, investigating multiple coagulation factors enhances the comprehensiveness of the analysis. The inclusion of middle‐aged women provides a relatively homogeneous population at risk of stroke but presumably without a large effect of age‐related cardiovascular risk factors and atherosclerosis. The inclusion of four distinct patient groups—ischemic stroke only, migraine with aura only, both ischemic stroke and migraine, and neither—allowed for more detailed comparisons of coagulation factor levels across these groups.

Our results support the hypothesis that endothelial activation and dysfunction may be involved in migraine‐ischemic stroke pathophysiology, underscoring the need for further research into these mechanisms as potential therapeutic targets for early stroke risk detection in women with migraine. The optimal prevention of stroke in people with migraine remains uncertain. While managing vascular risk factors is currently the standard approach, the role of endothelial activation in stroke, particularly among women who smoke, requires further investigation. Future studies should also explore a broader range of endothelial biomarkers to better understand the migraine–stroke relationship.

## 5 Conclusion

Our findings suggest that endothelial activation, indicated by increased VWF:Ag levels, may be more relevant than a procoagulant state alone in the pathophysiology of ischemic stroke in patients with a history of migraine. We did not observe an association between coagulation factors and WMH volume, suggesting other mechanisms may be involved in their development.

## Author Contributions

**Mariam Ali:** conceptualization, writing – original draft, visualization, formal analysis, software, project administration. **Nelleke van der Weerd:** conceptualization, writing – review and editing, investigation, project administration, resources. **Hendrikus J. A. van Os:** conceptualization, methodology, writing – review and editing, project administration, resources. **Annelise E. Wilms:** project administration, resources, methodology, data curation. **Ghislaine Holswilder:** writing – review and editing, project administration, data curation, investigation, methodology. **Katie M. Linstra:** writing – review and editing, conceptualization, investigation, methodology, project administration, data curation, resources. **Thijs W. van Harten:** investigation, methodology, writing – review and editing, project administration, data curation, resources, software. **Suzanne C. Cannegieter:** supervision, formal analysis, visualization, methodology, writing – review and editing. **Bob Siegerink:** methodology, visualization, writing – review and editing, formal analysis, supervision. **Bart J. M. van Vlijmen:** writing – review and editing, visualization, investigation, resources, data curation. **L. Renee Ruhaak:** investigation, visualization, writing – review and editing, data curation, resources. **Nyika D. Kruyt:** visualization, supervision, writing – review and editing. **Mark C. Kruit:** investigation, methodology, validation, writing – review and editing, supervision, data curation, resources. **Antoinette Maassen Van Den Brink:** conceptualization, investigation, funding acquisition, methodology, validation, visualization, writing – review and editing, project administration, data curation, supervision, resources. **Gisela M. Terwindt:** conceptualization, investigation, funding acquisition, methodology, validation, visualization, writing – review and editing, data curation, resources, supervision, project administration, writing – original draft. **Marieke J. H. Wermer:** conceptualization, investigation, funding acquisition, methodology, validation, visualization, writing – review and editing, supervision, resources, data curation, writing – original draft, project administration.

## Conflicts of Interest

G.M.T. reports independent support from the Dutch Research Council, DHF, Dutch Brain Foundation, Dioraphte, Clayco Foundation and consultancy for Novartis, Lilly, Teva, Abbvie, Organon, Pfizer, and Lundbeck. A.E.W. and G.M.T. report grant support from the European Community (101070917), Stichting Dioraphte (20010407), and Clayco Foundation. A.M.V.D.B. received honoraria and research/travel grants from Allergan/AbbVie, Amgen/Novartis, Eli Lilly, Manistee, Pfizer, Satsuma, Teva, and Tonix as well as independent support from the Dutch Research Council and ZonMw. T.W.H. received support from DHF and NWO under the joint strategic research program, “Earlier recognition of cardiovascular diseases.”

## Supporting information


**Data S1.** Supplemental Methods and Appendix: CREW consortium member list.

## Data Availability

The data that support the findings of this study are available from the corresponding author upon reasonable request.
