# Supplementary material for: Coagulation Factors and White Matter Hyperintensities in Middle‐Aged Women With and Without Migraine and Ischemic Stroke
Source: Eur J Neurol. 2025 Feb 28;32(3):e70063. doi: 10.1111/ene.70063 (PMC11871085; doi:10.1111/ene.70063)
Supplement: Supplementary file 1 — Data S1. Supplemental Methods and Appendix: CREW consortium member list. [file ENE-32-e70063-s001.docx]

**SUPPLEMENTAL MATERIAL**

**Supplemental Methods**

Image acquisition

The scanning protocol included the following sequences: 3D T1-weighted (repetition time (TR)/echo time (TE) 8.2/4.5 ms, voxel size 1.00 x 1.00 x 1.00 mm), 2D T2-weighted (TR/TE 4783/80 ms, voxel size 0.43 x 0.50 x 3.00 mm), and 3D Fluid Attenuated Inversion Recovery (FLAIR) images (TR/TE 4800/302 ms, inversion time 1650 ms, matrix size 1.12 x 1.12 x 1.12 mm).

White matter hyperintensities (WMH) volume assessment

WMHs were defined as areas with intensities 2.8 SD greater than the mean on bias field-corrected FLAIR images, in regions normally containing only white matter.(1) In ischemic stroke patients, WMH volume was scored in the unaffected hemisphere and multiplied by two for comparison with group 2 (migraine with aura) and group 4 (no history of stroke or migraine). WMHs were considered present if they appeared hyperintense on FLAIR and were categorized as periventricular, deep, or cerebellar. Lesions were classified as periventricular if contiguous with the margins of the lateral ventricles, and as deep if entirely separate from the lateral ventricles.

Quantitative protein mass spectrometry

In this study, fibrinogen and Factor IX (FIX) were quantified using a semiautomated laboratory-developed multiplex mass spectrometry (MS) test on 1290 Infinity II ultra-high-performance liquid chromatography instrument coupled to 6495 triple quadrupole-MS (Agilent Technologies, Santa Clara, USA) instrumentation. This strategy allows for direct and multiplexed quantitation of coagulation proteins.(2) Sample preanalysis was performed on a 96-channel Agilent BRAVO automated liquid handling platform (Agilent Technologies) according to a validated protocol previously established for the quantitative measurement of apolipoproteins in serum and plasma,(3, 4) with minor adjustments: Plasma samples were diluted twenty-fold in 100 mmol/L ammonium bicarbonate at pH 8.1. Stable isotope-labeled (SIL) peptides were added as internal standards to ensure accurate quantification and were specific to the proteins of interest (peptides were synthesized by the Peptide and Tetramer Facility Immunology, Leiden University Medical Center, the Netherlands). Proteins were then denatured, alkylated, and digested with trypsin.(3, 4) The digestion conditions were optimized to ensure stable peptide formation for accurate protein quantitation.

Following digestion, the peptides were desalted using solid-phase extraction (SPE) with Oasis PRiME HLB μElution Plates (Waters, Wexford, Ireland) and subsequently dried, reconstituted in a mobile phase, and analyzed by liquid chromatography-mass spectrometry (LC-MS). Peptides were separation on a ZORBAX SB-C18 column (Agilent Technologies) with a 32-minute gradient. Mass spectrometry acquisition was performed in multiple reaction monitoring (MRM) mode using positive ionization mode and 9 peptides were monitored for fibrinogen and 2 peptides for FIX. Data integration and analysis were conducted using Agilent MassHunter Workstation and RStudio. External calibration was performed using five native human citrate plasmas, which were value assigned with indirect traceability to WHO standards in g/L for fibrinogen and IU for FIX. Samples were analyzed in four batches, each containing four internal quality controls (IQC). The IQC passed predefined specifications with CVs ranging between 1.6% and 2.3% for fibrinogen and 2.5% and 5.2% for FIX.

**CREW member list**

The CREW consortium consists of (in alphabetical order):

Yolande Appelman^1^, Sara Baart^2,3^, Laura Benschop^2,3^, Eric Boersma^2^, Laura Brouwers^3,4^, Ricardo Budde^2^, Suzanne Cannegieter^5^, Veerle Dam^3,6^, Rene Eijkemans^6^, Bart Fauser^4^, Michel Ferrari^5^, Arie Franx^3^, Christianne de Groot^1^, Marlise Gunning^3,4^, Annemieke Hoek^7^, Erik Koffijberg^6,8^, Wendy Koster^2^, Mark Kruit^5^, Giske Lagerweij^3,6^, Nils Lambalk^1^, Joop Laven^2^, Katie Linstra^2,3,5^, Aad van der Lugt^2^, Angela Maas^9^, Antoinette Maassen van den Brink^2^, Cindy Meun^2,3^, Saskia Middeldorp^10^, Karel GM Moons^6^, Bas van Rijn^4^, Jeanine Roeters van Lennep^2^, Jolien Roos-Hesselink^2^, Luuk Scheres^3,10^, Yvonne T. van der Schouw^6^, Eric Steegers^2^, Regine Steegers^2^, Gisela Terwindt^5^, Birgitta Velthuis^3^, Marieke Wermer^5,7^, Bart Zick^2,5^, Gerbrand Zoet^3,4^

^1^Amsterdam UMC – location VUmc, Amsterdam, the Netherlands

^2^Erasmus MC University Medical Center, Rotterdam, the Netherlands

^3^Netherlands Heart Institute, Utrecht, the Netherlands

^4^University Medical Center Utrecht, Utrecht, the Netherlands

^5^Leiden University Medical Center, Leiden, the Netherlands

^6^Julius Center, Utrecht, University Medical Center, Utrecht, the Netherlands

^7^University Medical Center Groningen, Groningen, the Netherlands

^8^University of Twente, Enschede, the Netherlands

^9^Radboud University Medical Center, Nijmegen, the Netherlands

^10^Amsterdam UMC – location AMC, Amsterdam, the Netherlands

**References**

1. Tustison NJ, et al. N4ITK: improved N3 bias correction. IEEE Trans Med Imaging. 2010;29(6):1310-20.

2. Camilleri E, et al. Quantitative protein mass spectrometry for multiplex measurement of coagulation and fibrinolytic proteins towards clinical application: What, why and how? Thromb Res. 2024;241:109090.

3. Ruhaak LR, et al. Robust and Accurate 2-Year Performance of a Quantitative Mass Spectrometry-Based Apolipoprotein Test in a Clinical Chemistry Laboratory. Clin Chem. 2018;64(4):747-9.

4. van den Broek I, et al. Automated Multiplex LC-MS/MS Assay for Quantifying Serum Apolipoproteins A-I, B, C-I, C-II, C-III, and E with Qualitative Apolipoprotein E Phenotyping. Clin Chem. 2016;62(1):188-97.
